# Sequencing the Obligate Intracellular *Rhabdochlamydia helvetica* within Its Tick Host *Ixodes ricinus* to Investigate Their Symbiotic Relationship

**DOI:** 10.1093/gbe/evz072

**Published:** 2019-04-04

**Authors:** Trestan Pillonel, Claire Bertelli, Sébastien Aeby, Marie de Barsy, Nicolas Jacquier, Carole Kebbi-Beghdadi, Linda Mueller, Manon Vouga, Gilbert Greub

**Affiliations:** Center for Research on Intracellular Bacteria, Institute of Microbiology, Lausanne University Hospital, University of Lausanne, Switzerland

**Keywords:** chlamydia, HGT, comparative genomics, shotgun metagenomics, tick symbiont

## Abstract

The *Rhabdochlamydiaceae* family is one of the most widely distributed within the phylum *Chlamydiae*, but most of its members remain uncultivable. *Rhabdochlamydia* 16S rRNA was recently reported in more than 2% of 8,534 pools of ticks from Switzerland. Shotgun metagenomics was performed on a pool of five female *Ixodes ricinus* ticks presenting a high concentration of chlamydial DNA, allowing the assembly of a high-quality draft genome.

About 60% of sequence reads originated from a single bacterial population that was named “*Candidatus Rhabdochlamydia helvetica*” whereas only few thousand reads mapped to the genome of “*Candidatus Midichloria mitochondrii*,” a symbiont normally observed in all *I. ricinus* females. The 1.8 Mbp genome of *R. helvetica* is smaller than other *Chlamydia*-related bacteria. Comparative analyses with other chlamydial genomes identified transposases of the PD-(D/E)XK nuclease family that are unique to this new genome. These transposases show evidence of interphylum horizontal gene transfers between multiple arthropod endosymbionts, including *Cardinium* spp. (*Bacteroidetes*) and diverse proteobacteria such as *Wolbachia, Rickettsia* spp. (*Rickettsiales*), and *Caedimonas varicaedens* (*Holosporales*). Bacterial symbionts were previously suggested to provide B-vitamins to hematophagous hosts. However, incomplete metabolic capacities including for B-vitamin biosynthesis, high bacterial density and limited prevalence suggest that *R. helvetica* is parasitic rather than symbiotic to its host.

The identification of novel *Rhabdochlamydia* strains in different hosts and their sequencing will help understanding if members of this genus have become highly specialized parasites with reduced genomes, like the *Chlamydiaceae*, or if they could be pathogenic to humans using ticks as a transmission vector.

## Introduction

Ticks are the most common arthropod vector of human and animal diseases (Moutailler et al. 2016).They also frequently carry mutualist symbionts; all females and nearly 50% of the males of the European tick *Ixodes ricinus* carry the *Rickettsiales* symbiont “*Candidatus Midichloria mitochondrii*.” The high prevalence of this symbiont suggests an obligate association between the two species, but the role of the symbiont in the biology of *I. ricinus* remains unknown (Ahantarig et al. 2013; Moutailler et al. 2016). As confirmed by whole genome sequencing, ticks are incapable of de novo heme synthesis and acquire heme from exogenous source (Gulia-Nuss et al. 2016; Perner et al. 2016). Hence, tick symbionts such as *Coxiella*, *Rickettsia*, and *Francisella*-like may be involved in the provisioning of nutrients like B-vitamins and cofactors lacking from their exclusively hematophagous diet (Rio et al. 2016; Duron et al. 2018; Husnik 2018).

*Chlamydiae* DNA was identified in approximately 0.89% of *I. ricinus* ticks in Switzerland based on a large-scale screen of 62,889 *I. ricinus* ticks sampled from 172 different sites (Croxatto et al. 2014; Pilloux et al. 2015). Based on the 16S rRNA gene sequence of 359 positive samples, over 29% (105/359) belonged to the *Rhabdochlamydiaceae* family and exhibited high amounts of chlamydial DNA (up to 8 × 10^6^ copies/µl, see Pilloux et al. 2015). Additional studies reported the presence of *Rhabdochlamydia* spp. in various tick populations around the world (Hokynar et al. 2016; Burnard et al. 2017). However, no *Rhabdochlamydia* isolate has been cultivated from ticks yet. The two other described species, were also identified in arthropods, suggesting that *Rhabdochlamydia* spp. may infect a wide range of arthropods. “*Candidatus Rhabdochlamydia porcellionis*” was isolated from hepatopancreatic cells of the terrestrial crustacean *Porcellio scaber* (Kostanjsek 2004) and “*Candidatus Rhabdochlamydia crassificans*” was identified in the cockroach *Blatta orientalis* (Corsaro et al. 2007). Up to now, only “*Candidatus Rhabdochlamydia porcellionis*” could be cultured in SF-9 cells (Sixt et al. 2013). Both *R. porcellionis* and *R. crassificans* species exhibited a particular cell morphology; elementary bodies presented a five layered cell wall and oblong translucent structures in the cytoplasm (Corsaro et al. 2007; Kostanjsek 2004). Older records describe very similar bacteria with pentalaminar cell walls infecting the spider *Pisaura mirabilis* (Morel 1978), the scorpion *Buthus occitanus* (Morel 1976), and the midge larvae *Chironomus dorsalis* (Morel 1980). No hybridization between the DNA from the scorpion and the midge larvae parasites could be observed and Guanine–Cytosine (GC)-content and genome size estimations were significantly different (1,550 kb [kilobases], 41% GC vs. 2,650 kb, 36.7% GC, respectively) for these two putative chlamydia-related bacteria of arthropods, suggesting that they likely belong to different genera (Frutos et al. 1989, 1994). The proposition to classify these bacteria in the genus *Porochlamydia* (Morel 1976) was not recognized and *Porochlamydia* were considered to be part of the *Rickettsiella* genus (Fournier and Raoult 2005). More recent metagenomics analyses of the spider *Oedothorax gibbosus* microbiome revealed a high prevalence of *Rhabdochlamydia* spp. in one spider population (Vanthournout and Hendrickx 2015), suggesting that these so-called “*Porochlamydia*” might in fact represent two members of the *Rhabdochlamydiaceae* family.

If *R. porcellionis* and *R. crassificans* were shown to be detrimental to their host (Radek 2000; Kostanjšek and Pirc Marolt 2015), the nature of the relationship between *Rhabdochlamydia* spp. and ticks, as well as their pathogenicity toward humans, remain to be investigated. In the present study, we bypassed the difficult step of cell-culture by sequencing the genome of a *Rhabdochlamydia* within its host *I. ricinus*, allowing us to identify distinctive features and to learn about the biology of this largely unknown chlamydial family.

## Materials and methods

### Genome Sequencing, Assembly, and Annotation

A sample with five female ticks from Lucerne area (Switzerland) in the work of Pilloux et al. (2015) was selected for high throughput sequencing. Genomic DNA was extracted and purified using Wizard Genomic DNA purification kit (Promega, Duebendorf, Switzerland). Genomic libraries were constructed using Nextera XT library kit (Illumina), normalized on beads and pooled into a single library for 150 base pairs (bp) paired-end sequencing using the MiSeq (Illumina, San Diego, CA).

Illumina reads were filtered and trimmed with fastq-mcf 1.1.2 (Aronesty 2013), keeping 150 bp reads with an average Phred quality score higher than 30 (--max-ns 1 -l 150 -L 150 --qual-mean 30). Filtered reads were assembled with Edena 3 (Hernandez et al. 2008) and metaSPAdes 3.10.1 (Nurk et al. 2017) with k-mer or overlap size ranging from 51 to 127. In addition, reads that did not map on published genome assemblies (GCA_000973045.1 and GCF_000208615.1) from the ticks *I. ricinus* and *Ixodes scapularis* (Pagel Van Zee et al. 2007; Quillery et al. 2014) were assembled using MaSuRCA 2.1.9 (Zimin et al. 2013) and Velvet 1.2.09 (Zerbino and Birney 2008). Scaffolds were blasted against a database including the *I. ricinus* and *I. scapularis* genome assemblies with BlastN (e-value ≥ 1e^−^^5^, identity ≥ 80%) to remove contaminants. Scaffolds that did not show significant similarity to *Ixodes* sequences were further investigated as follows: (1) Coding sequences were predicted with prodigal 2.6 (parameters: -c -m -g 11 -p meta -f sco -q) (Hyatt et al. 2010). (2) Each predicted coding sequences (CDS) was assigned to a taxonomic rank with Kaiju 1.6.2 (Menzel et al. 2016) and the proGenomes database (Mende et al. 2017). (3) A bacterial phylum was assigned to each scaffold based on the most frequent phylum assigned to its predicted CDSs. The assembly graph produced by metaSPAdes was visualized with Bandage (Wick et al. 2015) in order to investigate the connectivity of scaffolds assigned to the *Chlamydiae* phylum. Finally, 8 assemblies from the 4 assemblers presenting between 107 and 66 scaffolds were compared and combined with Consed (Gordon et al. 1998). The combined assembly was split in case of disagreement between assembly methods. Only scaffolds larger than 1,000 bp exhibiting no significant similarity to *Ixodes* sequences, consistent GC-content and sequencing depth were retained for further analyses. The completeness of the final genome assembly was estimated based on the identification of 104 nearly universal single-copy genes with CheckM (Parks et al. 2015).

Raw reads were mapped to the assembled *Rhabdochlamydia helvetica* genome as well as to *I. scapularis*, *I. ricinus*, and “*Candidatus Midichloria mitochondrii*” (GCA_000973045.2, GCF_000208615.1, and GCF_000219355.1). The mapping was done with BWA mem version 0.7.17 (Li and Durbin 2009) with a minimum seed length of 23 and all 4 genomes combined into a single fasta file.

Scaffolds were reordered based on the reference genome of *Simkania negevensis* Z (NC_015713) using Mauve v. 2.3.1 (Darling et al. 2004). Genome annotation was performed using Prokka v. 1.10 (Seemann 2014). Domains were annotated using InterProScan 5.18-57 (Jones et al. 2014). Predicted coding sequences were compared with the COG database (Galperin et al. 2014) using BlastP 2.2.28+ (Camacho et al. 2009) with an e-value cutoff of 1e^−5^, and to the RefSeq database release 79 (Haft et al. 2018) using PLAST 2.3.1 (parameters: -a 8 -M BLOSUM62 -e 1e-3 -G 11 -E 1 -force-query-order 1000 -max-hit-per-query 100 -max-hsp-per-hit 1) (Van Nguyen and Lavenier 2009). The coding density was calculated based on predicted open reading frames (ORF), or published annotations. For draft assemblies, only contigs larger than 10 kb were used to estimate the coding density.

### Confirmation of Tick Species from Genomic Data

To confirm that the host species was *I. ricinus*, raw reads were mapped on the 18S rRNA sequence of 82 different tick species (merged into a single fasta file, supplementary table S1, Supplementary Material online). Mapped reads were extracted and remapped individually on each 18S rRNA sequence and inspected with IGV (Robinson et al. 2011).

### Metabolism and Respiratory Chains Annotation

Metabolic pathways and modules were investigated using the Kyoto Encyclopedia of Genes and Genomes (KEGG) database (Ogata et al. 1999). KEGG orthology assignments were done using the GhostKOALA web server (Kanehisa et al. 2016). Mapping between KEGG orthologs and pathways/modules was done using the KEGG API (http://www.kegg.jp/kegg/docs/keggapi.html; last accessed April 10, 2019). Specific focus was made on basic energy metabolism, amino acids, cofactors, and vitamins biosynthesis. Transporters were annotated using gBlast3 from the transporter classification database (Saier et al. 2016), with an e-value cutoff of 10^−5^ and 50% coverage of both query and hit. When proteins of interest could not be identified, pseudogenes remnants or unpredicted ORFs were searched in raw contigs of the *Rhabdochlamydia* assembly using TBlastN and in the raw reads using Diamond (Buchfink et al. 2015).

### Cluster of Orthologs and Phylogenetic Reconstructions

Protein sequences of 61 PVC (*Planctomycetes*, *Verrucomicrobia*, and *Chlamydiae*) superphylum strains, including 31 members of the *Chlamydiales* order, were downloaded from RefSeq (supplementary table S2, Supplementary Material online) and grouped into orthologous groups using OrthoFinder 0.4.0 with default parameters (Emms and Kelly 2015). The complete orthology table is reported in supplementary table S3 (Supplementary Material online). The genome assemblies of the “*Candidatus Limichlamydia*” strain SM23_39 (marine sediments metagenome) and “*Candidatus Hydrochlamydia*” strain Ga0074140 (drinking water treatment plant metagenome), were not considered for genome content analyses. Unannotated genome assemblies were annotated with Prokka v.1.10. In addition, all predicted protein sequences were annotated using the same tools as for *R. helvetica* (see above). Protein identities were calculated based on multiple sequence alignments built using MAFFT 7.058b (Katoh and Standley 2013). Gaps were not considered in pairwise identity calculations. Genome maps were generated using Circos (Krzywinski et al. 2009). A phylogenetic tree was reconstructed based on 172 conserved single-copy proteins with RAxML 8.2.0 (Stamatakis 2014) using the LG substitution matrix (PROTGAMMALG) with distinct partitions for each of the 172 alignments and 100 rapid bootstrap replicates. This model was preferred based on previous experience with similar data sets (Pillonel et al. 2015). Figures with phylogenies and associated data were drawn with the Python package ete2 (Huerta-Cepas et al. 2010).

Proteins harboring a PD-(D/E)XK nuclease family transposase domain (Pfam accession PF12784) were searched in 6661 reference and representative genomes (downloaded from RefSeq, September 2017) with hmmsearch (HMMER v. 3.1b2 [Eddy 2011] with “–—cut_tc” parameter). The 1,911 identified proteins were further filtered based on similarity to at least 1 of the 6 *R. helvetica* homologs (filtered with BlastP v. 2.7.1 with an e-value of 1e^−^^5^ and 80% of hsp query coverage) and clustered at 90% identity with cd-hit v. 4.6 (Fu et al. 2012) to reduce redundancy. The phylogeny was reconstructed with FastTree v.2.1.9 (Price et al. 2010) double precision with default parameters.

## Results and Discussion

### Sequencing the Endosymbiont Genome within Its Tick Host

Shotgun metagenomics of the tick pool DNA produced 25.7 million paired-ends reads in two sequencing runs. Among 80 tick species, *I. ricinus* and *I. scapularis* recruited the highest number of reads by mapping on the 18S rRNA (respectively 985 and 835). Because some regions of the 18S rRNA are strictly identical between ticks species, all recruited reads were mapped again individually on *I. scapularis* and *I. ricinus* sequences. The single base of 18S rRNA sequence enabling to distinguish both species was identical to *I. ricinus* variant, confirming that all five ticks in the pool indeed belonged to *I. ricinus* species. No other polymorphism could be identified except for one position exhibiting two populations of reads.

Sixteen million high-quality reads were used to assemble the genome de novo. Most raw reads mapped either to the *R. helvetica* assembly (59.96%) or to one of the two *Ixodes* genomes (40.03%). Only 8,087 reads mapped to “*Ca. M. mitochondrii*” genome. Scaffolds assembled de novo were carefully investigated to remove contaminants such as *Ixodes* sequences (low sequencing depth, GC-content >40%) (supplementary fig. S1, Supplementary Material online). All scaffolds larger than 12.5 kb were classified as belonging to the *Chlamydiae* phylum (Most of them have a median depth >1600x and a GC content <40%) (supplementary fig. S1, Supplementary Material online). Chlamydial scaffolds formed a highly interconnected network of about 1.88 Mbp in the assembly graph (supplementary fig. S2, Supplementary Material online). The final manually curated assembly of 38 scaffolds, comprising 1,830,543 bp, was estimated to be complete (104/104 CheckM markers identified) and exhibited no detectable contamination (no duplicated marker gene). The cumulative G + C skew of the reordered assembly exhibited the typical inverted “V” shape of bacterial genomes (supplementary fig. S3, Supplementary Material online). Altogether, these results indicate that *Rhabdochlamydia* DNA was largely dominant in the pool of ticks sequenced, and only few sequences from other bacterial species were obtained, allowing to assemble a high-quality draft genome. A putative 23,934 bp circular plasmid was identified thanks to its uniformly lower sequencing depth as compared with the rest of the assembly (supplementary fig. S4, Supplementary Material online). It encodes a homolog of the plasmid integrase pGP8-D commonly found in chlamydial plasmids.

### A New Species of the *Rhabdochlamydia* Genus

**Fig. 1.**
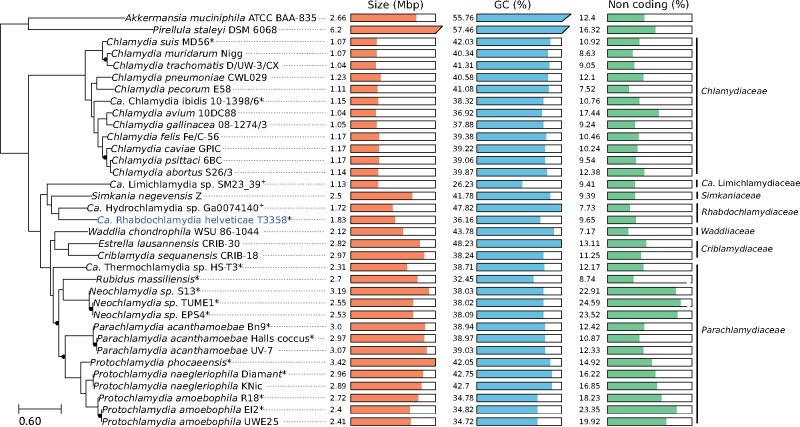
—Phylogeny and comparison of genome characteristics in the order *Chlamydiales*. The phylogeny was reconstructed using the Maximum Likelihood method implemented in RAxML. It was inferred based on the concatenated alignment of 172 single-copy protein sequences conserved in all genomes included in the phylogeny. Bootstrap supports lower than 100 are indicated with black dots. For draft genomes (indicated with asterisks), the proportion of noncoding sequences was estimated based on contigs larger than 10 kb. Plus symbols (+) indicate metagenome derived genome assemblies that were excluded from genome content analyses.

The 16S ribosomal sequence exhibited 97.88% and 97.98% identity with *R. porcellionis* (AY223862) and *R. crassificans* (AY928092), respectively. The conservation of this single gene is not sufficient to accurately classify *Chlamydiae* at the genus and species level (Pillonel et al. 2015). However, given the level of sequence divergence (>2%) and the lack of other *Rhabdochlamydia* genomes available, we considered this strain as a new Candidatus species named “*Candidatus Rhabdochlamydia helvetica*”. It exhibited 91.78% and 88.93% sequence identity respectively with the 16S and 23S rRNA of *S. negevensis* strain Z (supplementary table S4, Supplementary Material online). The reconstruction of the *Chlamydiae* phylogeny based on a concatenated set of 172 conserved protein sequences (fig. 1, supplementary table S5, Supplementary Material online) as well as the high number of orthologous protein families (supplementary figs. S4 and S5, Supplementary Material online) confirmed that *S. negevensis* is the closest sequenced relative of *R. helvetica.*

### Horizontal Gene Transfers Likely Occurred with Other Symbionts and Eukaryotic Hosts

**Fig. 2.**
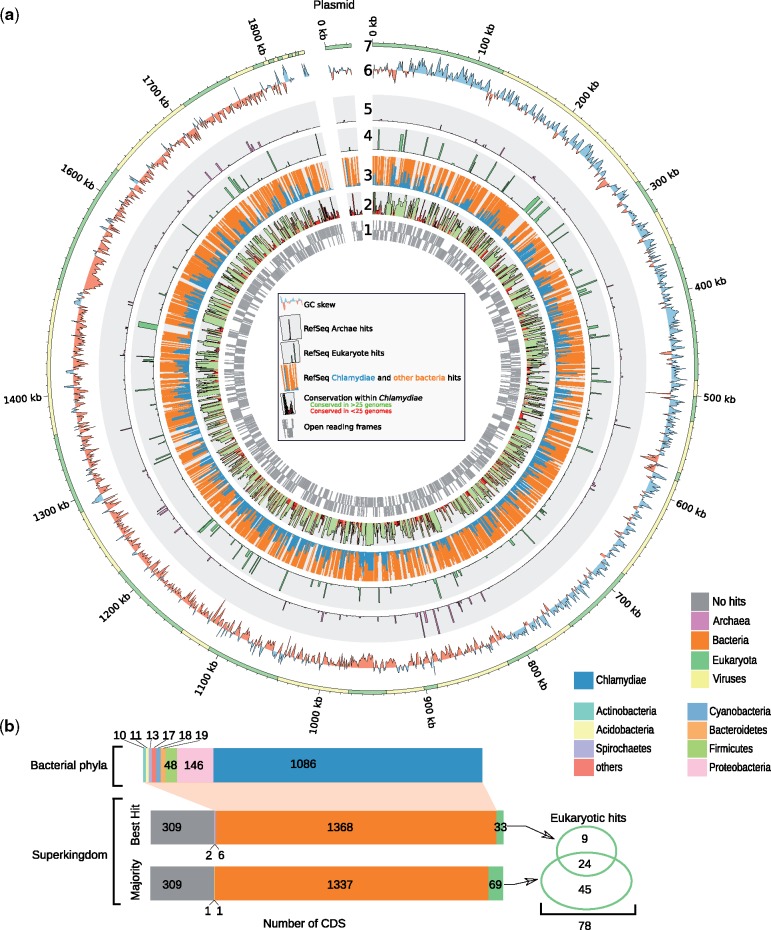
—(*a*) Protein homologs to the *R. helvetica* proteome in the National Center for Biotechnology Information (NCBI) RefSeq database. The inner gray circle (1) reports the localization of genes encoded on leading and lagging strands. The second circle (2) reports the conservation of the protein families in the 61 genomes included in the comparison (supplementary table S2, Supplementary Material online). Protein families conserved in <25 genomes are highlighted in red. The following circles report the taxonomic classification of the top 100 NCBI RefSeq hits of each *R. helvetica* protein: circle (3): number of chlamydial (blue) and other bacteria (orange) hits; circle (4): number of eukaryotic hits (green); circle (5): number of archaeal hits (pink). The “two outer circles” report (6) the GC-skew along the genome and (7) the localization of gaps in the genome assembly. (*b*) Summary of RefSeq hits taxonomy. Left part: consensus taxonomy of the top 100 best RefSeq hits (majority rule) and taxonomy of the best RefSeq hit of each *R. helvetica* protein. Right part: number of proteins with significant similarity to eukaryotic sequences.

Over 62.8% of the 1,717 predicted proteins of *R. helvetica* exhibited a best RefSeq hit within the *Chlamydiae* phylum (Fig. 2). Comparative analyses of 31 chlamydial genomes from 6 families (supplementary table S1, Supplementary Material online, excluding the 2 metagenome-assembled genomes) revealed that 322 (18%) *R. helvetica* CDS exhibited no ortholog in other chlamydial genomes (supplementary fig. S4, Supplementary Material online, purple CDS). Among these, 73 had significant hits in RefSeq database (supplementary table S6, Supplementary Material online). Six are PD-(D/E)XK nuclease family transposases exhibiting more than 60% sequence identity with proteins of *Occidentia massiliensis*, a soft tick symbiont from the *Rickettsiaceae* family (Mediannikov et al. 2014). The transposase phylogeny including homologs identified in 6,661 RefSeq reference and representative genomes (supplementary fig. S6, Supplementary Material online) showed that the 6 *R. helvetica* homologs are monophyletic and cluster with phylogenetically diverse intracellular bacteria belonging to *Bacteroidetes* (*Cardinium* spp.) and *Proteobacteria* in the orders *Rickettsiales* (*Wolbachia*, *Rickettsia*, *Orientia*, and *Occidentia* spp.), *Holosporales* (*Caedimonas varicaedens*), and *Myxococcales* (*Pajaroellobacter abortibovis*). Highly similar sequences were also identified in two arthropod genomes (*Cimex lectularius* and *Vollenhovia emeryi*). This could represent an example of interkingdom horizontal gene transfer (HGT), but might also result from the contamination of the two arthropod genomes with DNA sequences of colonizing bacteria. Indeed, *C. lectularius* was shown to carry *Wolbachia* symbionts (Rasgon and Scott 2004). Interphylum transfer of transposases is not uncommon, particularly in case of shared habitats (Hooper et al. 2009), and was already observed in arthropod symbionts (Duron 2013). Several other proteins specific to *R. helvetica* showed significant sequence similarity to other obligate endosymbionts (supplementary table S6, Supplementary Material online). These results suggest the occurrence of HGTs with other bacteria sharing a similar niche, in this case ticks and other arthropods, as previously described for *Parachlamydia* and other ameba-infecting microorganisms (Gimenez et al. 2011).

Seventy-eight *R. helvetica* proteins exhibited significant similarity to eukaryotic sequences (fig. 2, supplementary table S7, Supplementary Material online). These include known cases of chlamydial HGT such as multiple enzymes involved in the metabolism of C5 isoprenoid (*ispD*, *ispG*, *ispE*) (Lange et al. 2000), menaquinone (*menA*, *menD*), uridine monophosphate (*pyrE*, *pyrF*, discussed below), heme (*hemY*), and Pimeloyl-ACP (*fabI*, *fabF*) (Collingro et al. 2011). As previously shown (Schmitz-Esser et al. 2010), proteins with bacteria-host interaction domains such as ankyrin repeats, ubiquitin protease or BTB/POZ domains also exhibit high proportions of BLAST hits in eukaryotic sequences.

**Fig. 3.**
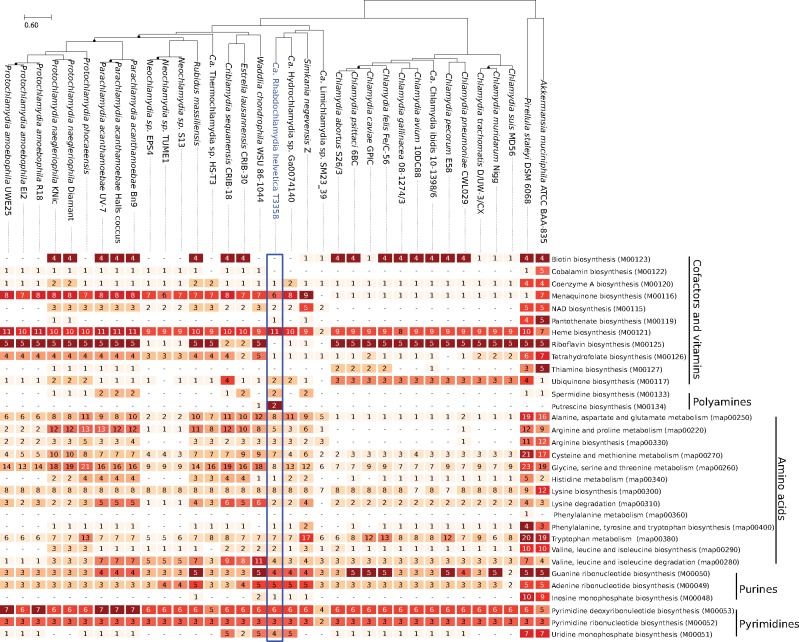
—Comparative analysis of cofactors, vitamins, amino acids, polyamines, and nucleotides metabolism. Each row reports the number of unique KEGG ortholog(s) for the corresponding module/pathway. NAD: nicotinamide adenine dinucleotide.

Among the 78 proteins sharing similarity with eukaryotic sequences (fig. 2), 17 proteins are unique to *R. helvetica*. A “methyl-accepting chemotaxis” domain-containing protein presented more than 50% amino acid identity with arthropod sequences. Two lysine/ornithine decarboxylases (including a partial 145 amino-acids sequence) participating to the polyamine biosynthesis pathway (supplementary fig. S7, Supplementary Material online) exhibited 60.25% identity with a sequence from the common house spider *Stegodyphus mimosarum* (see phylogeny in supplementary fig. S8, Supplementary Material online). Polyamines are essential for cellular growth of both prokaryotes and eukaryotes, and may be involved in bacterial pathogenesis (Shah and Swiatlo 2008), but their role in *Rhabdochlamydia* biology remains unknown. The *R. helvetica* genome encodes all enzymes necessary for the biosynthesis of polyamines from l-arginine and l-methionine (fig. 3, supplementary fig. S7, Supplementary Material online). All enzymes show evidence of HGT with eukaryotes (*rocF*, *speC*, supplementary figs. S8 and S9, Supplementary Material online) and proteobacteria (*metK*, *speD*, and *speE*, supplementary figs. S10–S12, Supplementary Material online), which suggests again a strong impact of HGT in endosymbiont evolution (Bertelli and Greub 2012; Alsmark et al. 2013; Clarke et al. 2013).

Intriguingly, two enzymes of the tricarboxylic acid cycle (TCA) cycle and the subunit A of the V-type ATPase exhibited a best RefSeq hits with coding sequences of the ameba *Acytostelium subglobosum*. A comparison of the entire *A. subglobosum* genome assembly showed that about 70 kb of scaffold 25 exhibits strong similarity to *Chlamydiae* proteins, indicating that the assembly is probably contaminated with a fragment of an unknown chlamydial symbiont genome (supplementary fig. S13, Supplementary Material online).

### Reduced Biosynthesis Capabilities Limit the Possible Mutualism with the Tick Host

Tick symbionts were previously suggested to provide essential nutrients to their host, however the metabolic repertoire of *Chlamydia-*related bacteria and *Chlamydia* sp. is known to be limited (fig. 3) (Omsland et al. 2014). Major differences comprise the presence of a glucokinase (RhT_00001) and a complete citric acid cycle. *R. helvetica* is able to synthesize aspartate (RhT_00062), asparagine, and glutamate and can further convert serine into glycine. Alanine production might be possible through the conversion of cysteine, but no alanine dehydrogenase could be identified. Noteworthy, no tryptophan operon—an important regulator of the chlamydial development cycle—could be identified although it is present in *S. negevensis* (Collingro et al. 2011; Omsland et al. 2014) and partially present in some genito-urinary strains of Chlamydia *trachomatis* (Omsland et al. 2014). An operon encoding the full pathway for the synthesis of pyrimidines could be identified, similar to those found in *W*addlia *chondrophila* and *Criblamydia sequanensis* (supplementary fig. S14, Supplementary Material online) (Bertelli et al. 2010, 2015). The closest RefSeq homologs of proteins encoded in the operon belong to Eukaryotes such as *Streptophyta* and *Arthropoda* (*pyrE*, *pyrF*, supplementary figs. S15 and S16, Supplementary Material online), amebae *(pyrD*, supplementary fig. S17, Supplementary Material online), and gamma-proteobacteria such as *Rickettsiella* spp., obligate intracellular bacteria infecting a wide range of arthropods (*pyrC, pyrB, carB, carA*, supplementary figs. S18–S21, Supplementary Material online), suggesting that the operon may have been acquired through HGTs. Biosynthesis of vitamins and cofactors is restricted to heme and menaquinone (Figure 3), suggesting that *R. helvetica* could participate to the supply—albeit limited—of some essential factors to its host like other symbionts of blood-feeding parasite (Leclerque 2008; Duron et al. 2018; Husnik 2018). However, *R. helvetica* is not able to synthetize quite a number of vitamins and cofactors, limiting its possible mutualistic interactions with the host, and rather suggesting a pathogenic behavior.

### Hallmarks of Chlamydial Parasitism

As mentioned in a previous publication and like all *Chlamydiae* sequenced to date, the genome of *R. helvetica* encodes homologs of the type three secretion system (T3SS) apparatus and chaperones (Pillonel et al. 2018). Homologs of several known T3SS effectors such as NUE, pkn5, and Lda2 were also identified (see supplementary table S8, Supplementary Material online, for detailed list of identified loci). Like its closest sequenced relative *S. negevensis*, *R. helvetica* does not encode a homolog of the protease-like activity factor (CPAF), a well-known chlamydial virulence factor. *R. helvetica* encodes about half as many transporters as *S. negevensis* (supplementary table S9, Supplementary Material online). It does not have a homolog of the sulfate permease (family 2.A.53) present in most chlamydial genomes nor the sodium-driven transporters found in *Chlamydiaceae* such as the anion/Na^+^ symporter SodiTl (2.A.47, CT_204), the amino acid symporters BrnQ (2.A.26, CT_554), and TnaT (CT_231). The loss of such transporters might be linked to the loss of the complex 1 of the respiratory chain involved in Na^+^ gradient generation. No homolog of the Na^+^/H^+^ antiporter NhaE (2.A.111: CT_805) was identified, but *R. helvetica* has a CPA2 family Na^+^/H^+^ antiporter homolog absent from the *Chlamydiaceae*. These transporters involved Na^+^ tolerance, pH homeostasis, tolerance to alkali, and fluctuations in osmolarity are infrequent in intracellular symbionts and pathogens (Krulwich et al. 2009). Some oligopeptide and amino acid transporters were identified in *R. helvetica*; A d-alanine/glycine sodium symporter (GlyP), an alanine sodium symporter (AgcS), two putative proton/glutamate-aspartate symporters (family 2.A.23), and an active oligopeptide transporter OppABCDF operon. The 13 periplasmic solute-binding proteins (OppA) constitute the largest group of paralogs in the *R. helvetica* genome.

Phosphorylated glucose can be imported through the UhpC transporter, a highly conserved transporter encoded in all sequenced chlamydia genomes (2.A.1.4.6). Like in other *Chlamydiae*, no phosphoenolpyruvate transport system was found in *R. helvetica*. Multiple cofactor and vitamin transporters were identified, including putative biotin transporters with low similarity to *Vibrio cholerae*, and three transporters with low similarity to riboflavin transporters from *Ochrobactrum anthropi* and *V. cholerae* (22%–28% identity). An S-adenosylmethionine transporter and a two-partner secretion operon with low similarity to a heme/hemopexin transport system (*huxA*/*huxB*) from *Haemophilus influenzae* were also identified (supplementary table S8, Supplementary Material online).

Seven ATP/ADP antiporter homologs were identified. One homolog (RhT_00329) is most closely related to *S. negevensis* SnNTT1 and *C. trachomatis* Npt1 which were shown to transport adenosine triphosphate (Adenosine diphosphate) and ATP/NAD, respectively (Knab et al. 2011; Fisher et al. 2013). Another homolog (RhT_01503) is more closely related to SnNTT2, a guanine nucleotide/ATP/H^+^ transporter (Knab et al. 2011). Three subsequent paralogs (RhT_00750, RhT_00751, RhT_00752) are phylogenetically most closely related to SnNTT4, whose substrate remains undetermined. The last putative ATP/ADP antiporters (RhT_00462, RhT_00945) are distant from all transporters characterized so far.

In summary, despite the presence of a larger metabolic repertoire than *Chlamydiaceae*, *Rhabdochlamydia* is probably highly dependent on its host, thus questioning the mutualistic or parasitic nature of the relationship with its host.

#### Rhabdochlamydia helvetica *Membrane Proteins*

The membrane of *Chlamydia* is thought to be stabilized by extensive disulfide bonds between the major outer membrane protein (MOMP) and two cysteine-rich proteins OmcB and OmcA (Hatch 1996). These three abundantly expressed proteins are the major constituents of the chlamydial outer membrane complex that also contains porins and polymorphic membrane proteins (Pmps) (Birkelund et al. 2009; Liu et al. 2010). The outer membrane protein composition of *Chlamydia*-related bacteria is very divergent, probably reflecting differences in their ecological niches, but all of them, with the notable exception of *S. negevensis*, contain OmcB, OmcA, and other cysteine-rich proteins (Collingro et al. 2011; Rusconi et al. 2013; Aistleitner et al. 2015). OmcB and OmcA could not be retrieved in the genome of *R. helvetica* that also encode few cysteine-rich membrane proteins of the OmpA family compared with *W. chondrophila* (*n* = 11) or Estrella *lausannensis* (*n* = 10) (Bertelli et al. 2010, 2015). However, the *R. helvetica* genome encodes one homolog of MOMP (RhT_00042) and five MOMP-like proteins (RhT_00150, RhT_00151, RhT_00153, RhTp_00022, RhT_01103). These latter have a 1.4%–2.7% higher cysteine content than the 37 *S. negevensis* MOMP and MOMP-like proteins (<1.1% cysteine) (Aistleitner et al. 2015). Like *S. negevensis* (Vouga et al. 2016), the membrane of *R. helvetica* might not be stabilized by a network of highly cross-linked proteins hence conserving more flexibility.

*Chlamydiaceae* bacteria express between 9 and 21 Pmps autotransporters that share little sequence similarity but play important roles in adhesion via conserved multiple repeats of the tetrapeptide motifs GGA(ILV) and FXXN (Mölleken et al. 2010; Becker and Hegemann 2014). The *R. helvetica* genome contains three *Simkania* PmpB homologs predicted to form a beta barrel structure in the outer membrane (RhT_00712, RhT_00713, and RhT_01040), but only RhT_01040 contains the GGAI sequence and none displays a FXXN motif. However, *R. helvetica* possesses one additional protein (RhT_00552) with an autotransporter beta-domain displaying similar structural features as the Pmp-like protein of *W. chondrophila* (wcw_0271); a signal peptide, a passenger domain, and a beta-barrel C-terminal domain. This protein containing five FXXN repeats and one repeat of the GGA(ILV) motif in the passenger domain could, by similarity with *wcw_0271*, be implicated in adhesion to the host cells (Kebbi-Beghdadi et al. 2015).

## Conclusion

The 1.83 Mb genome of *R. helvetica* sequenced directly from a pool of ticks provides the first molecular data to investigate the biology of this widespread genus (Lagkouvardos et al. 2014) infecting a wide range of arthropods (Kostanjsek 2004; Corsaro et al. 2007; Pilloux et al. 2015; Vanthournout and Hendrickx 2015; Cooling et al. 2017). Evidence of multiple HGTs between *Rhabdochlamydia*, arthropods and several lineages of arthropod symbionts suggests that *Rhabdochlamydiaceae* are widely distributed arthropod parasites and highlights the importance of nonvertical inheritance in the evolution of symbionts and parasites. Although arthropods are frequently engaged in mutualistic associations with bacteria (Moran et al. 2008), the *R. helvetica* genome only shows reduced metabolic capacities limiting any potential mutualism. In contrast, it encodes several ATP/ADP transporters and multiple transporters of vitamins, cofactors, amino acids, and oligopeptides. Considering that the 2.65 Gb genome of *I. scapularis* (Cramaro et al. 2017) is over 1,000-fold larger than the *R. helvetica* genome and that 60% percent of the raw reads obtained by shotgun metagenomics belonged to *R. helvetica*, the bacterial density was likely extremely high and not without consequences for the host. Altogether, the limited metabolic capacities, high bacterial density and low prevalence suggest that *R. helvetica* is rather parasitic to its host.

### Data Availability

The genome assembly of *R. helvetica* as well as raw sequencing reads are available under the European Nucleotide Archive project accession PRJEB24578.

## Acknowledgments

This project was funded by the budget of the Center for Research on Intracellular Bacteria. The salary of some of the authors (Carole Kebbi-Beghdadi, Marie de Barsy and Manon Vouga) are from diverse Swiss National Science Foundation grants.

## Supplementary Material

Supplementary data are available at *Genome Biology and Evolution* online.

## Supplementary Material

Supplementary Data
