# Supplementary material for: Sequencing the Obligate Intracellular Rhabdochlamydia helvetica within Its Tick Host Ixodes ricinus to Investigate Their Symbiotic Relationship
Source: Genome Biol Evol. 2019 Apr 4;11(4):1334–44. doi: 10.1093/gbe/evz072 (PMC6490308; doi:10.1093/gbe/evz072)
Supplement: Supplementary Data [file evz072_supp.zip › supplements.pdf]

Supplementary data: Sequencing the obligate intracellular *Rhabdochlamydia helvetica* within its tick host *Ixodes ricinus* to investigate their symbiotic relationship

Trestan Pillonel[1], Claire Bertelli[1], Sébastien Aeby[1], Marie de Barsy[1], Carole Kebbi-Beghdadi[1], Linda Mueller[1], Manon Vouga[1], and Gilbert Greub[1]

[1]*Center for Research on Intracellular Bacteria, Institute of Microbiology, Lausanne University Hospital, University of Lausanne, Switzerland*

March 2019

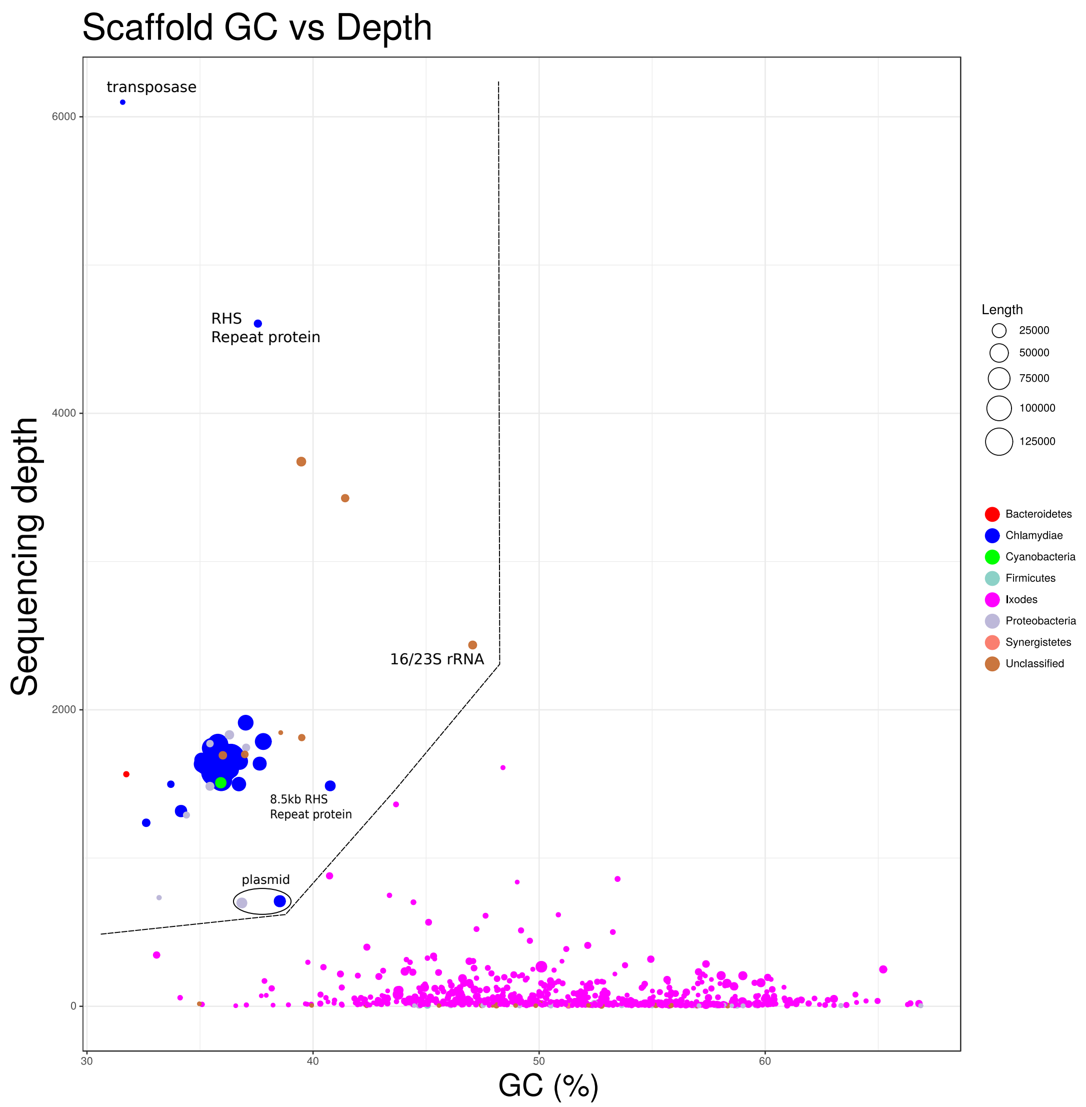

[h]

Figure S1: GC versus coverage plot of scaffolds assembled by the metaSpades assembler. Colors indicate the taxonomy of the scaffolds deduced from blastn against two *Ixodes* genomes and Kaiju hits (see method). *Ixodes* sequences exhibit higher GC content and much lower sequencing depth, allowing to discriminate the two genomes. All contigs larger than 12.5kb exhibit a majority of best Kaiju hits with chlamydial proteins. Taxonomic affiliation of smaller sequences is less precise, but the assembly graph (Figure S2) and additional comparative analyses (main manuscript) both support that the overwhelming majority of sequenced DNA (about 60% of the reads) belongs to the new chlamydial species. The black line separate sequences that were included into the final assembly from sequences excluded from the assembly (right part).

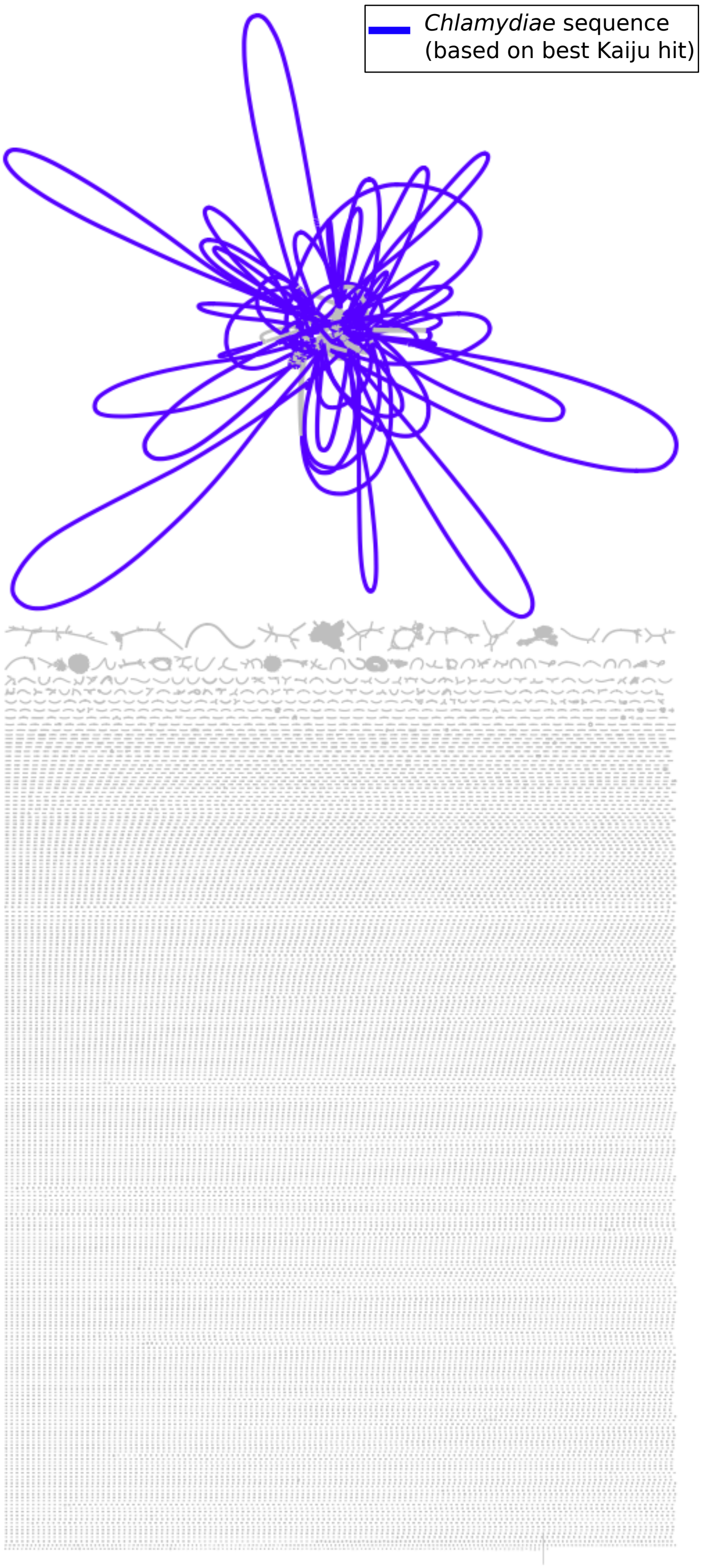

Figure S2: Visualization of the complete assembly graph produced by metaSPAdes with the Bandage software. Nodes classified as part of *Chlamydiae* based on best Kaiju hits are highlighted in blue. All contigs larger than 12.5kb are classified as part of the *Chlamydiae* phylum and form of a highly interconnected graph of about 1.88 Mbp.

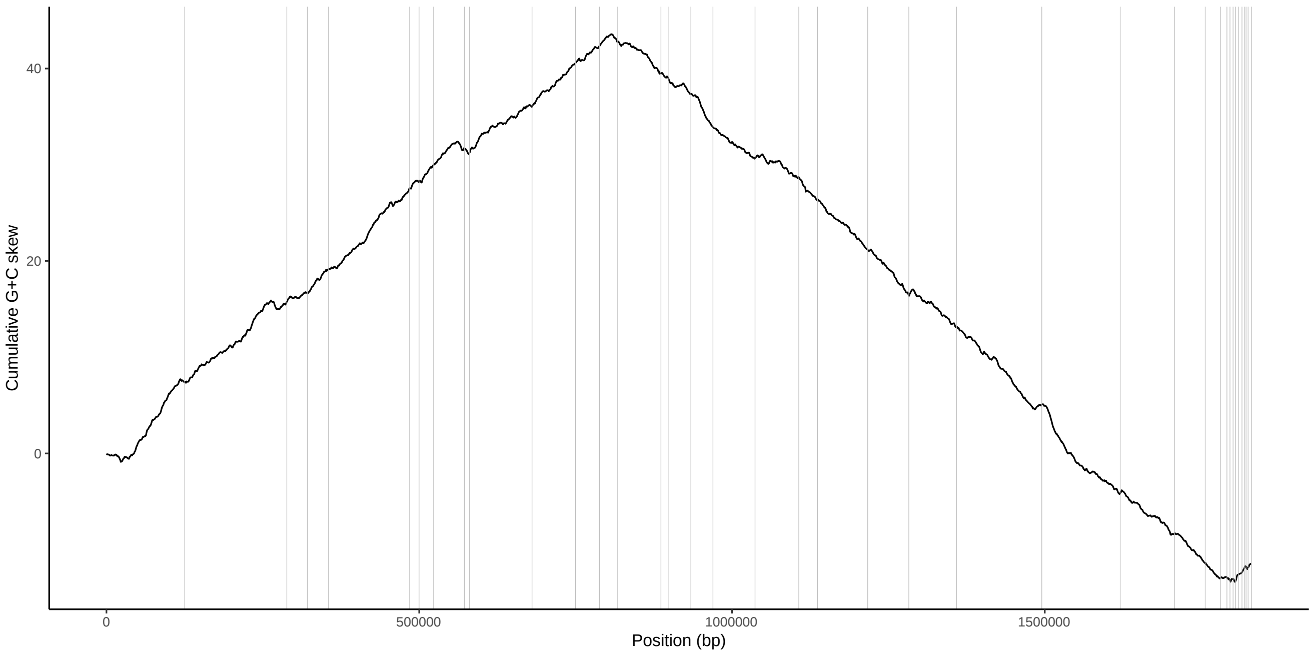

Figure S3: Cumative G+C skew of the *Rhabdochlamydia helvetica* assembly. Verical lines indicate scaffold limits. Scaffolds were ordered based on the complete genome of *Simkania negevensis*. It exhibits the typical inverted ”V” shape of bacterial genomes.

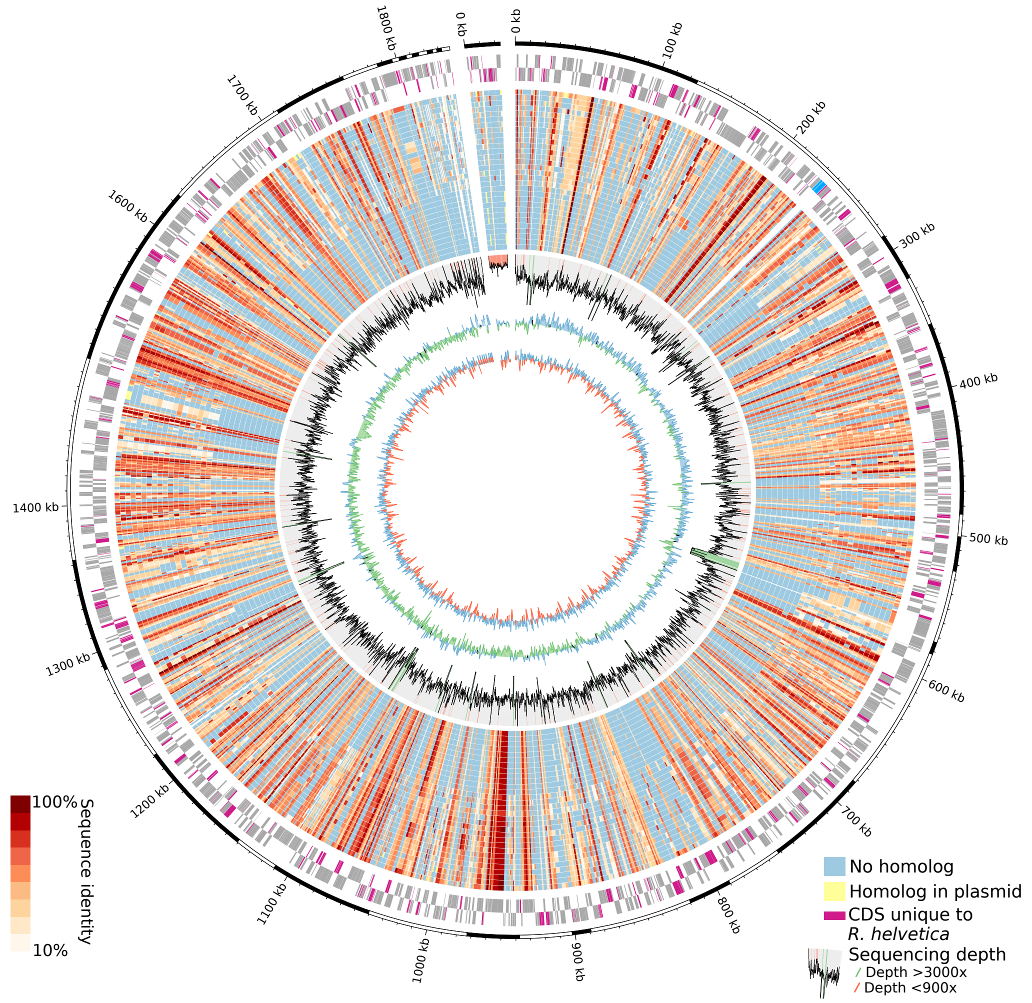

Figure S4: *R. helvetica* genome. The black and white outer circle reports contig boundaries after ordering against the complete genome of *S. negevensis*. The second and third circles indicate open reading frames (ORFs) on the leading and lagging strands, respectively. Pink ORFs highlight coding sequences that are unique to *R. helvetica*. Blue ORFs indicate rRNA genes. Inner blue/red circles indicate the presence (red scale indicating the percentage of identity to the closest ortholog) or absence (light blue) of ortholog(s) in other chlamydial genomes (see ordered genome list in Table S10). Homologs encoded on chlamydial plasmids are highlighted in yellow. The inner grey/red histogram reports the sequencing depth. Regions exhibiting a sequencing depth lower than 900 folds are highlighted in red. The putative plasmid (separate contig at the top) exhibits a uniformly lower coverage as compared to the chromosome. Several ORFs encoded on the plasmid have homologs encoded on known chlamydial plasmids (highlighted in yellow in the 30 compared genomes). Regions exhibiting a sequencing depth higher than 3000 are highlighted in green. The largest high depth regions located around 560kb and 1100kb are both repeat containing RHS (rearrangement hotspot) proteins. Smaller regions showing the highest depth are all transposases. The two last inner circles indicate the GC skew (blue for positive and green for negative) and GC content (blue for above-average and red for below-average).

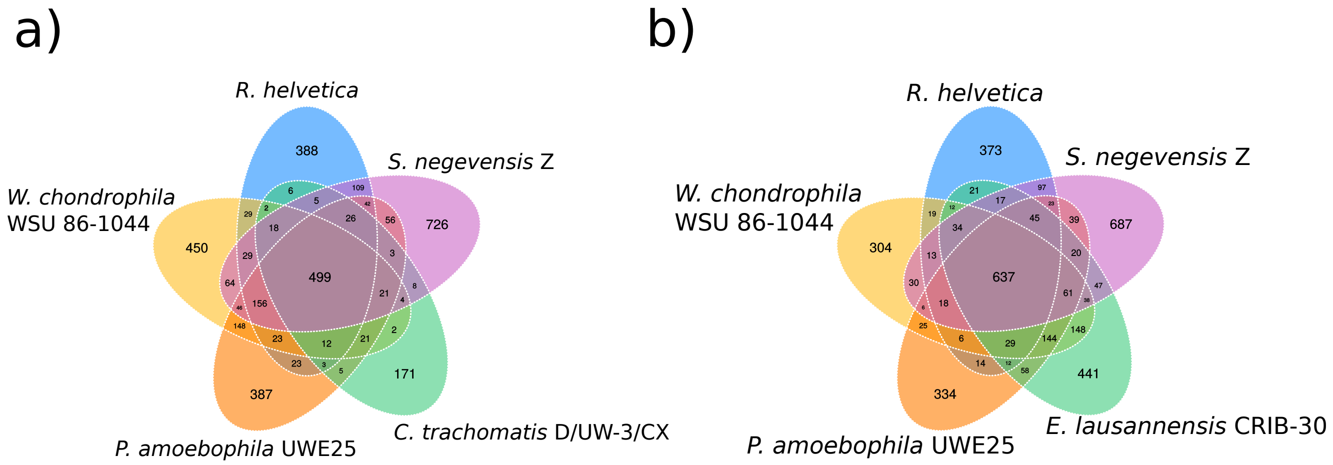

Figure S5: Venn diagram of shared orthologous groups between representatives of six family level lineages. A) Comparison of the *Rhabdochlamydia* genome with representatives of the *Waddliaceae*, *Parachlamydiaceae*, *Simkaniaceae* and *Chlamydiaceae* B) Comparison of the *Rhabdochlamydia* genome with representatives of 4 *Chlamydia*-related families. Proteins were clustered into orthologous groups with orthoFinder based on all-vs-all BLASTp.

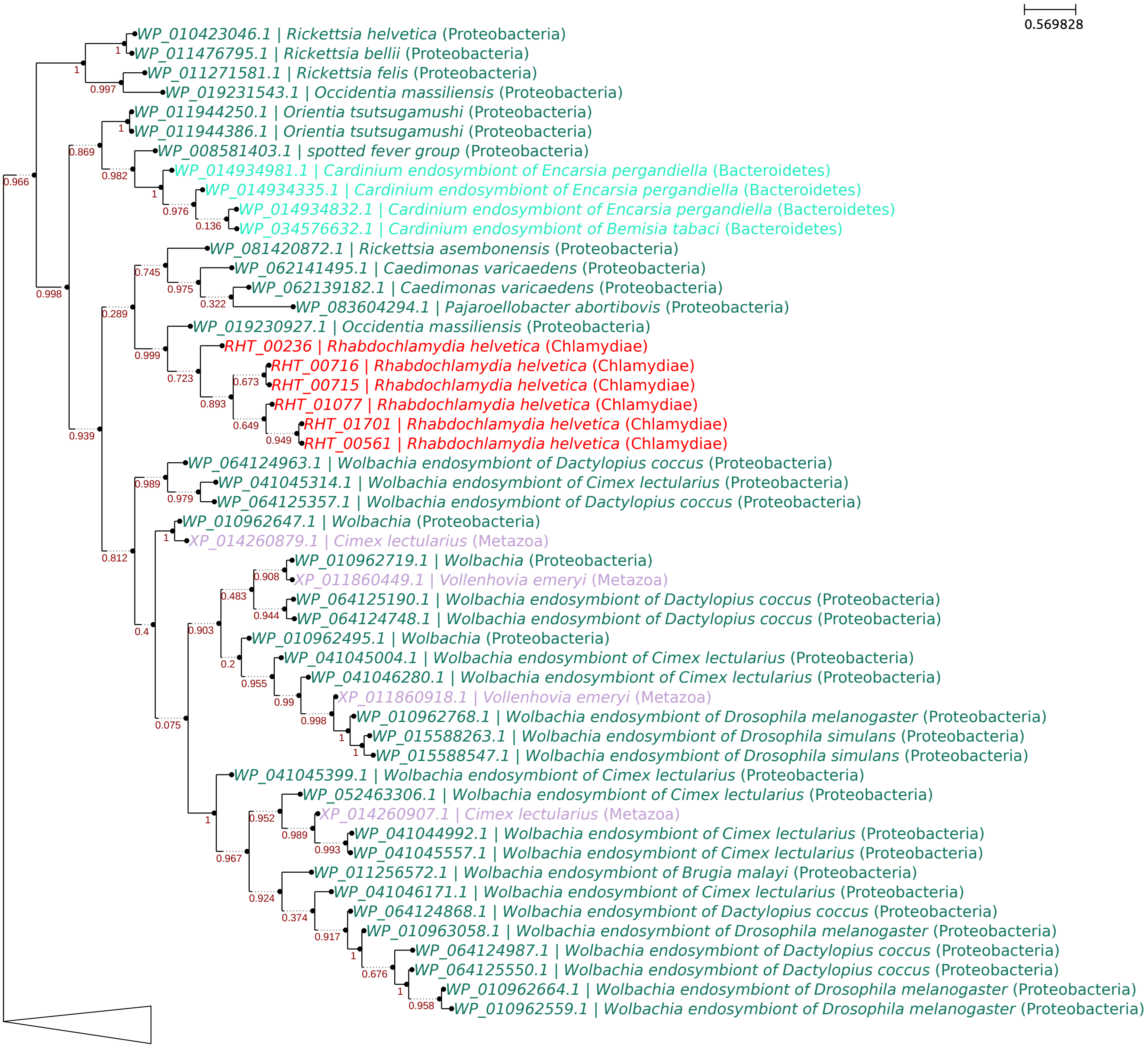

Figure S6: Phylogeny of PF12784 domain proteins. Of the 1911 RefSeq protein harboring the PF12784 domain, 538 were significantly similar to *R. helvetica* homologs (based on BLASTp with an e-value cutoff of 1e-5 and a minimum of 80% of query coverage). The phylogeny was reconstructed based on the non redundant set of 415 protein sequences. The tree was cut to show the closest identified homologs of the six *R. helvetica* proteins. Colours distiguish species from three bacterial phyla and metazoan sequences (written in parentheses). The two metazoan species are arthropods that might carry bacterial endosymbionts.

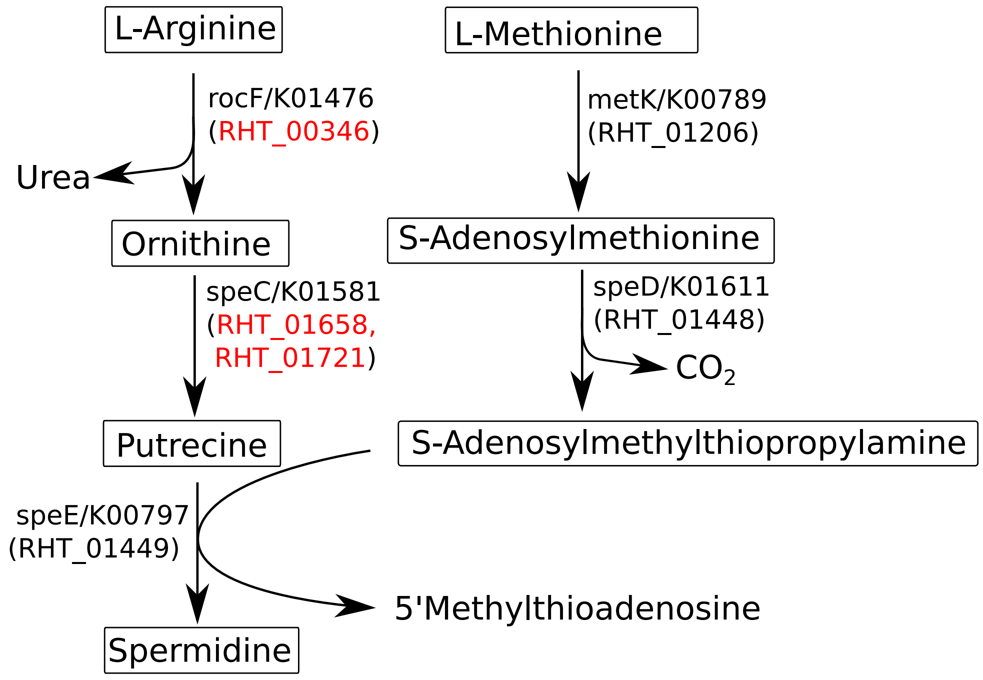

Figure S7: Polyamine biosynthesis pathway. The loci in red show evidence of horizontal gene transfer from the nematode *Caenorhabditis remanei* (RHT_00346) and the common house spider *Parasteatoda tepidariorum* (RHT_01658, RHT_01721). *metK*, *speD* and *speE* are most closely related to proteobacterial sequences.

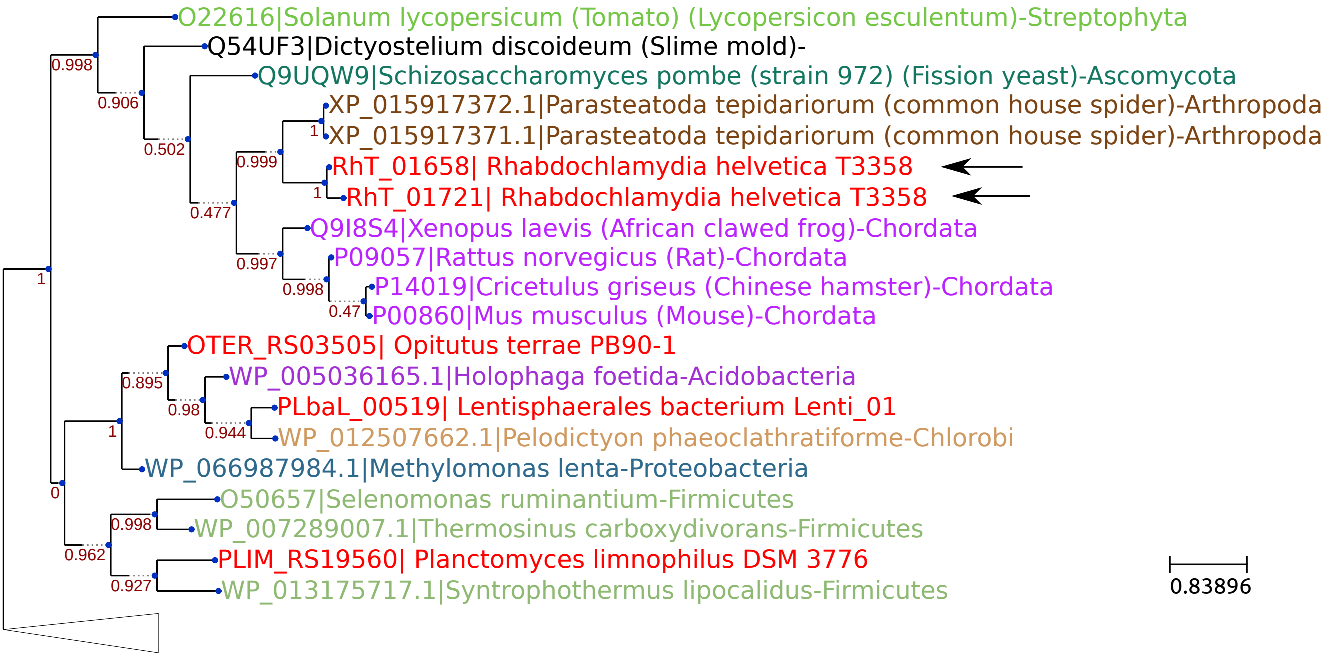

Figure S8: Phylogeny of the two Lysine/ornithine decarboxylases (*speC*) identified in *R. helvetica* genomes including closest identified RefSeq homologs.

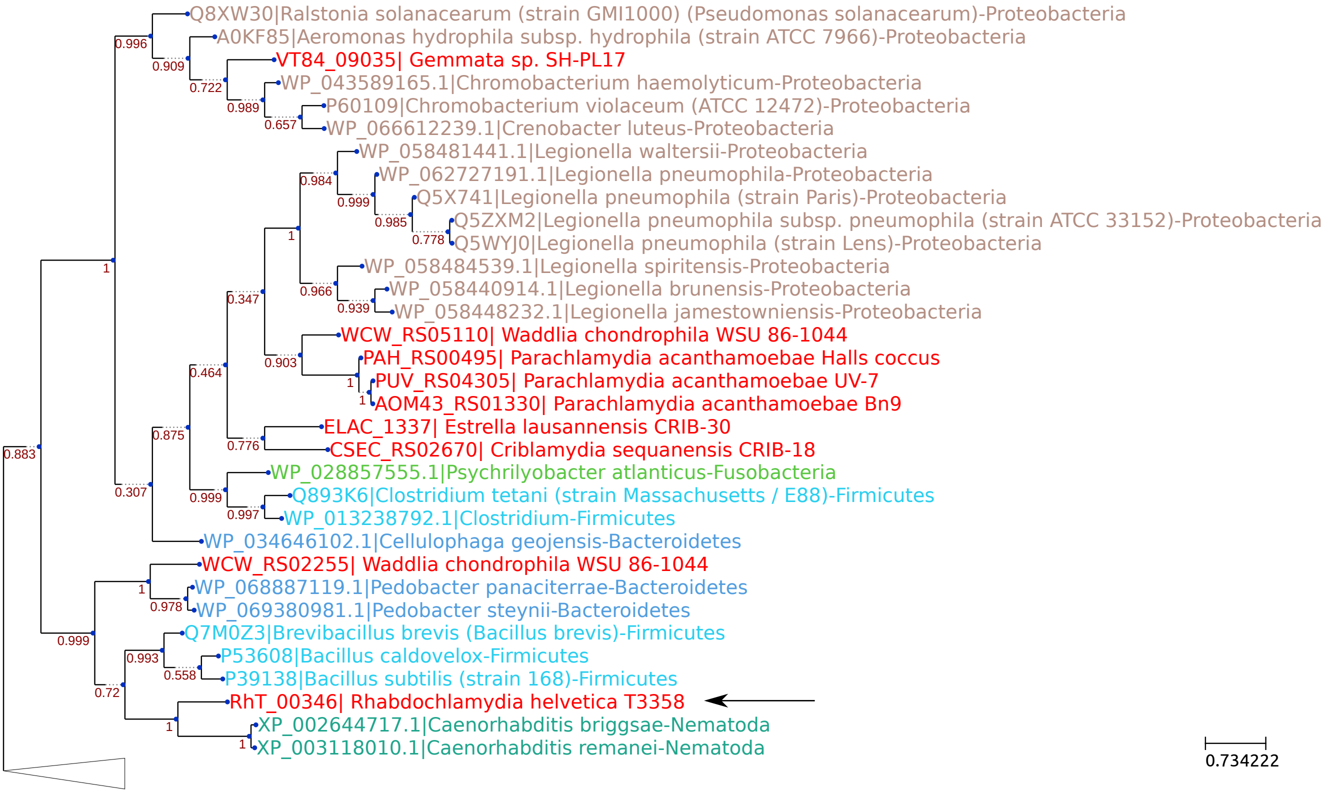

Figure S9: Phylogeny of *rocF* including closest identified RefSeq homologs.

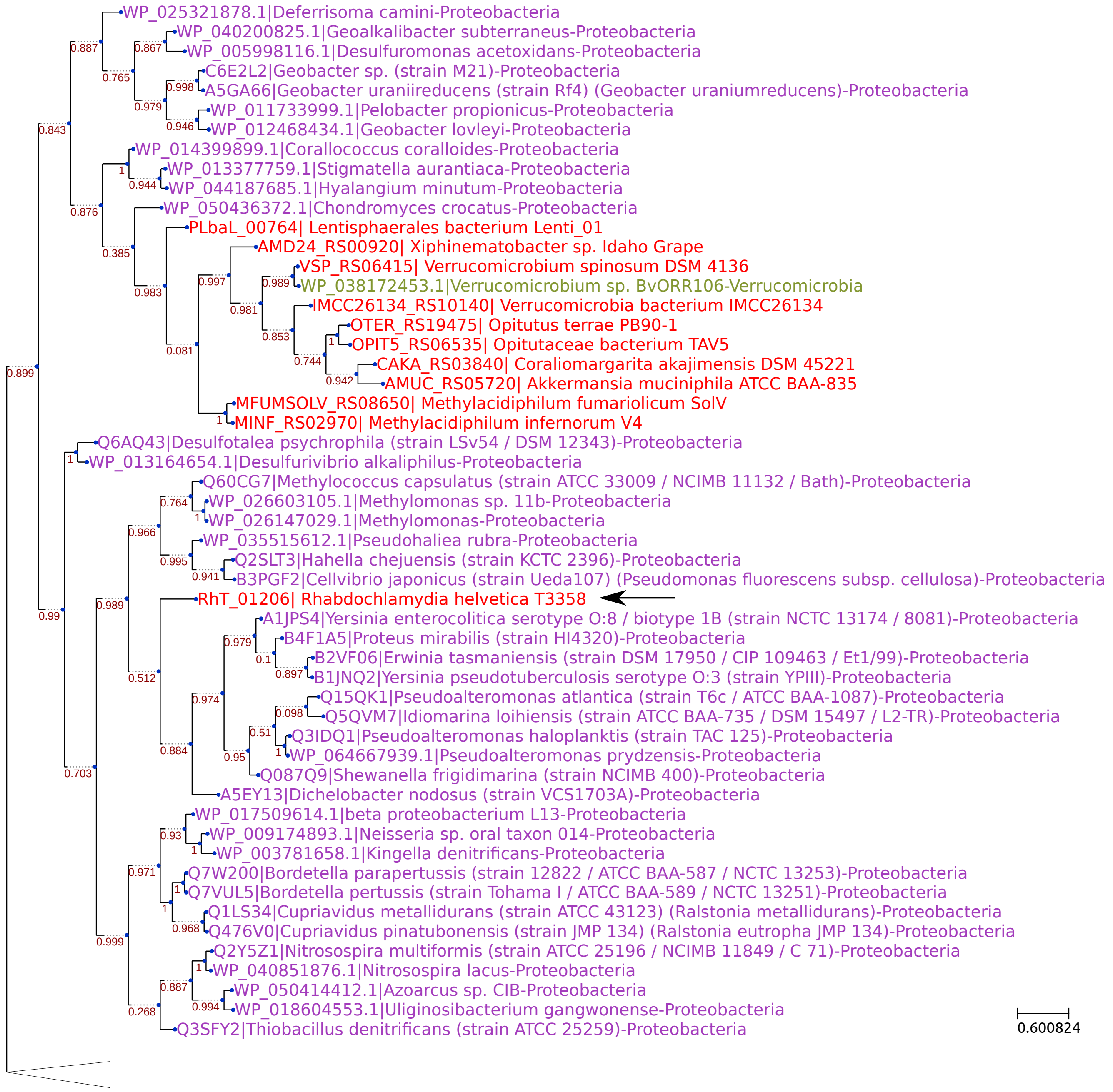

Figure S10: Phylogeny of *metK* including closest identified RefSeq homologs.

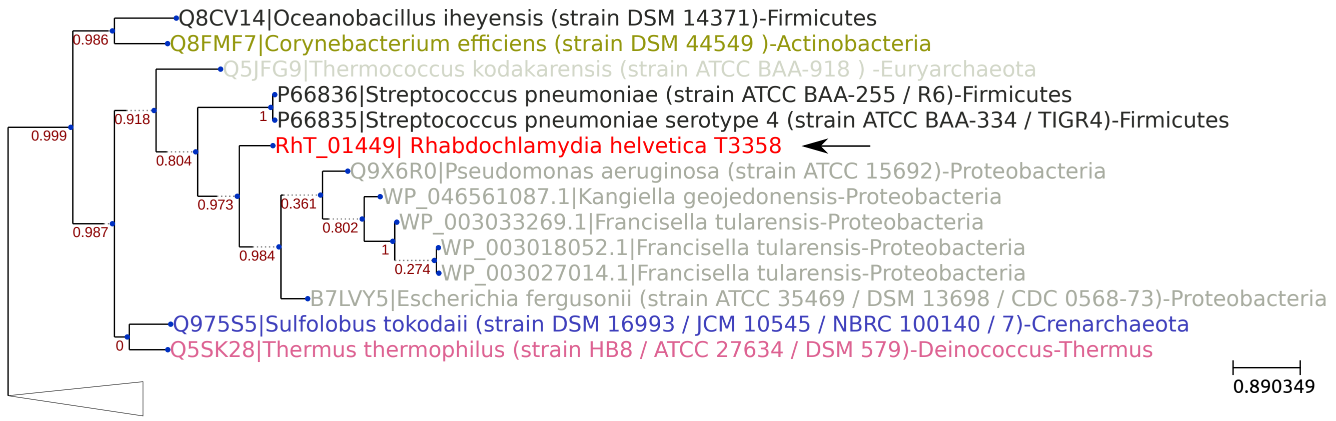

Figure S11: Phylogeny of *speE* including closest identified RefSeq homologs.

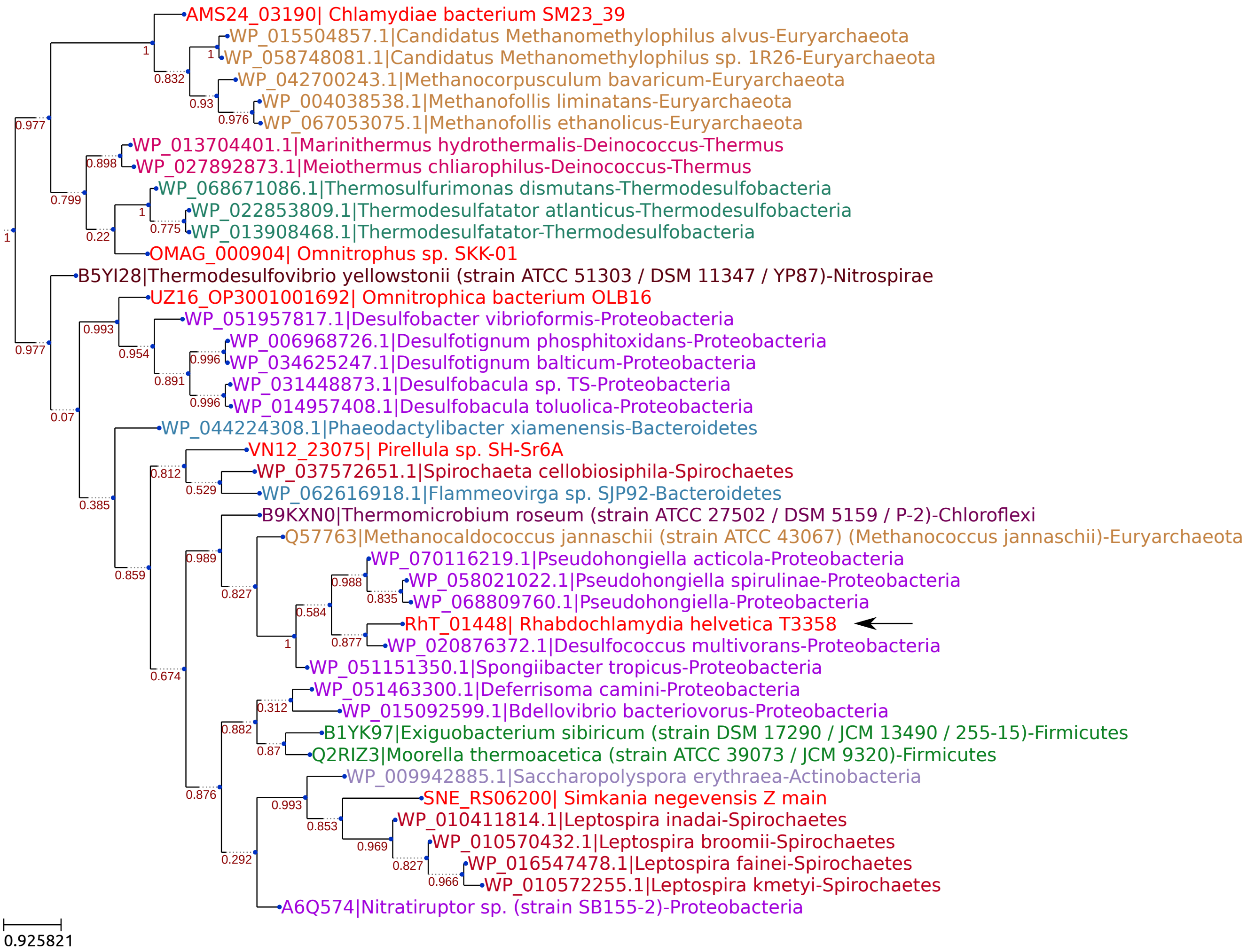

Figure S12: Phylogeny of *speD* including closest identified RefSeq homologs.

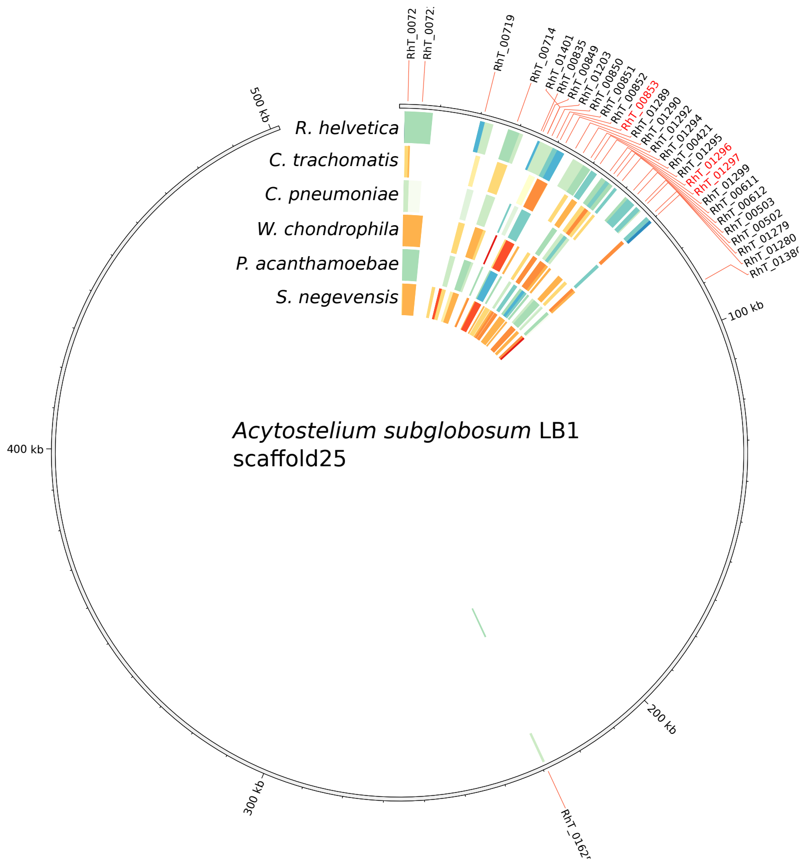

Figure S13: Evidence of contamination by an unknown *Chlamydiae* species in the genome assembly of *Acytostelium subglobosum*. Comparaisons of the genome of representative *Chlamydiae* with the scaffold 25 of the genome assembly of *A. subglobosum* suggest that a fragment of about 70 kilobases is of chlamydial origin. Couloured bars indicate aligned genomic regions. The alignment was done with promer (MUMmer software). *R. helvetica* locus tags reported in the outer circle exhibit more than 50% identity with the scaffold (Assembly accession: GCF_000787575).

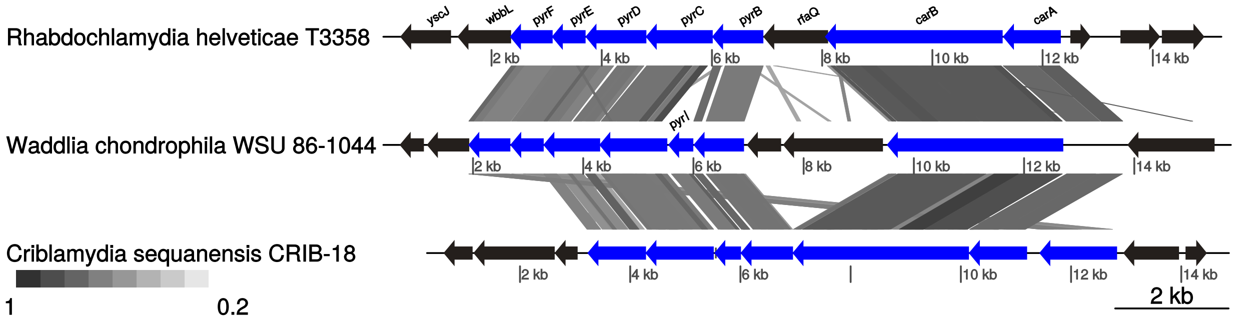

Figure S14: Uridine monophosphate biosynthesis operon. Conserved in *W. chondrophila*, *C. sequanensis* and *Candidatus* Hydrochlamydia sp. Ga0074140. *carA*, carbamoyl-phosphate synthase, small subunit; *carB*, carbamoyl-phosphate synthase, large subunit; *pyrB*, aspartate carbamoyltransferase; *pyrC*, dihydroorotase; *pyrD*, dihydroorotate dehydrogenase; *pyrE*, orotate phosphoribosyltransferase; *pyrF*, orotidine 5-phosphate decarboxylase; *pyrH*, uridylate kinase; *ndk*, nucleoside diphosphate kinase; *pyrG*, CTP synthase.

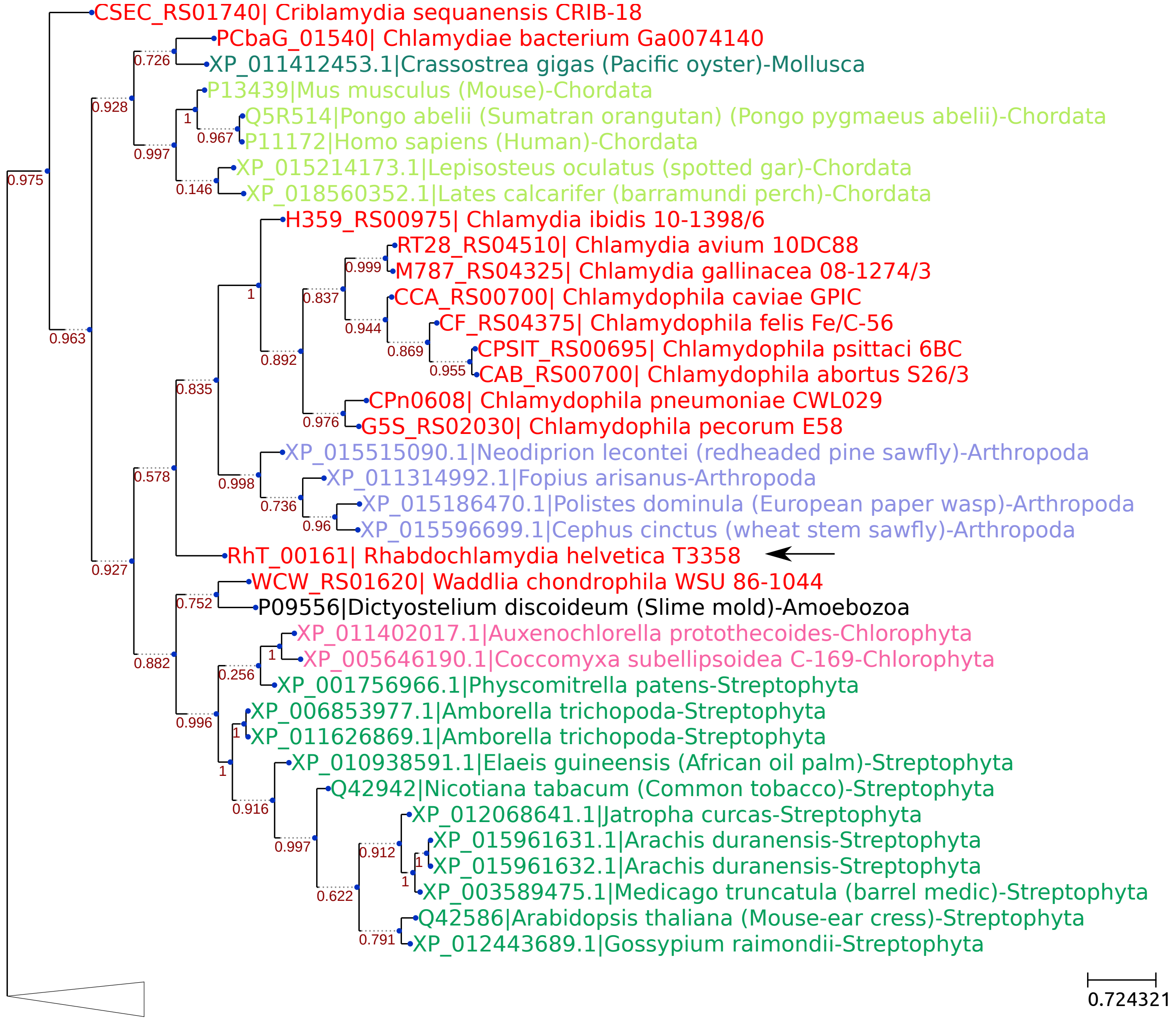

Figure S15: Phylogeny of *pyrE* including closest identified RefSeq homologs.

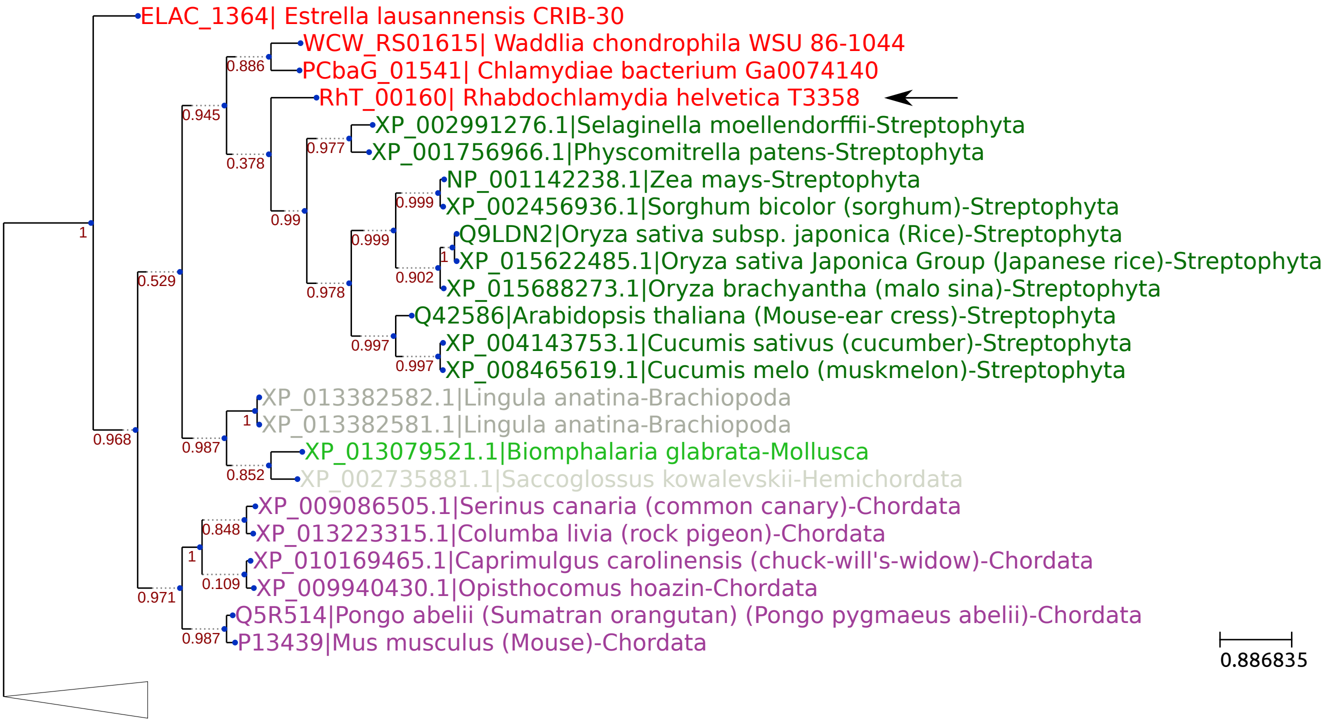

Figure S16: Phylogeny of *pyrF* including closest identified RefSeq homologs.

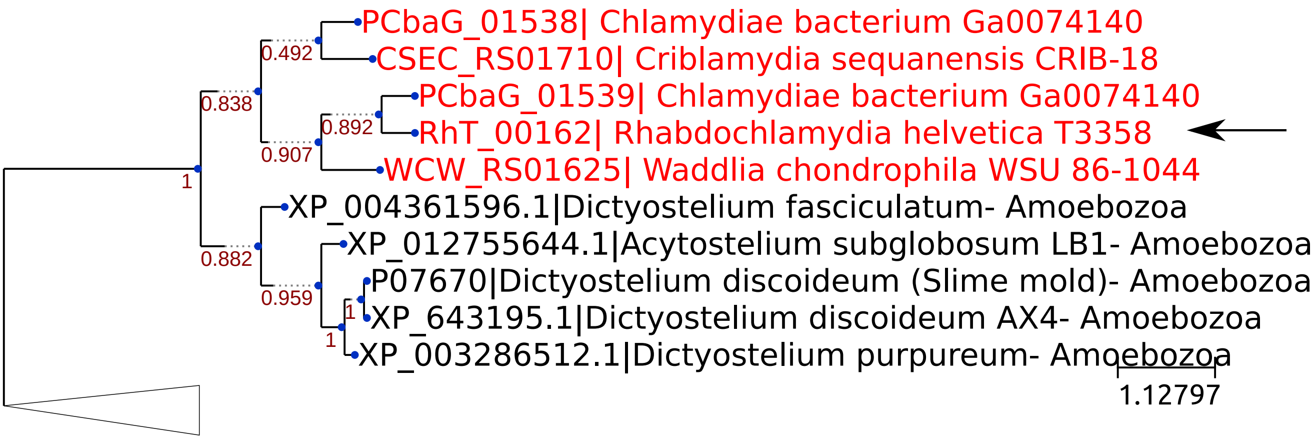

Figure S17: Phylogeny of *pyrD* including closest identified RefSeq homologs.

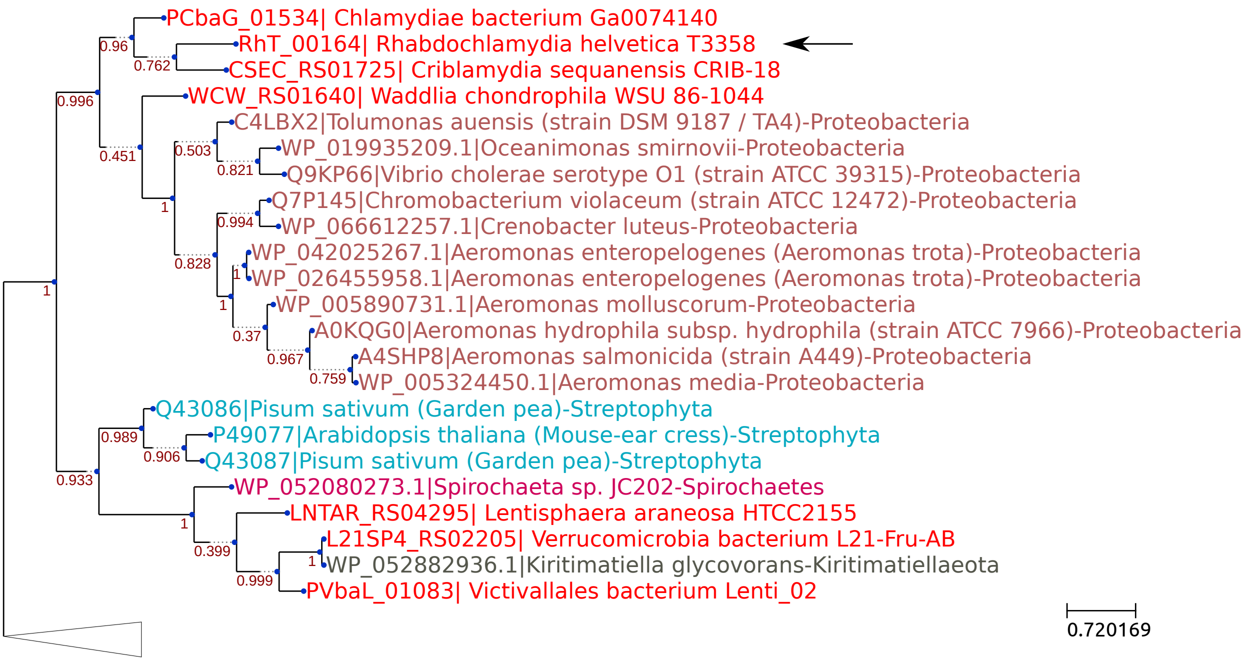

Figure S18: Phylogeny of *pyrB* including closest identified RefSeq homologs.

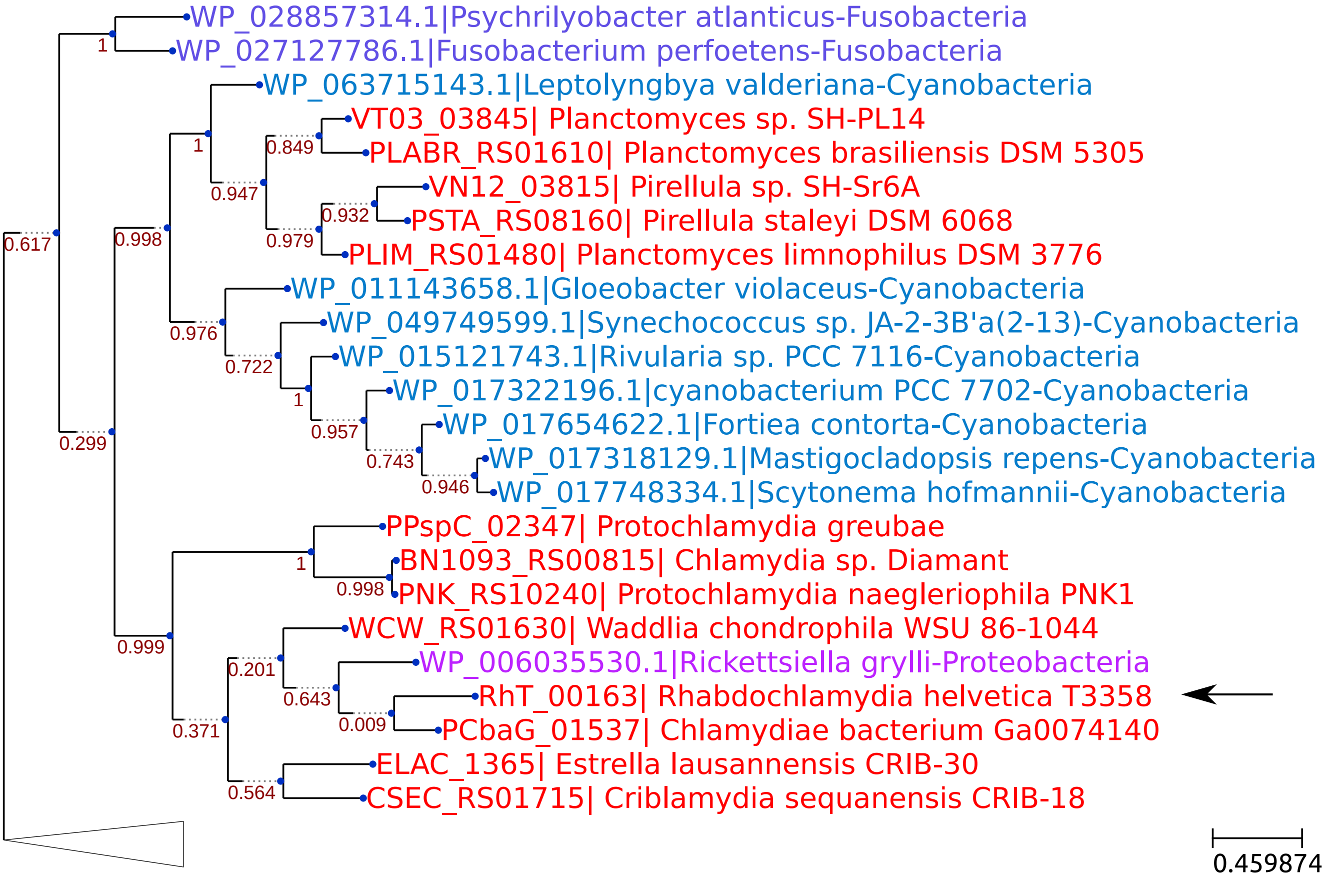

Figure S19: Phylogeny of *pyrC* including closest identified RefSeq homologs.

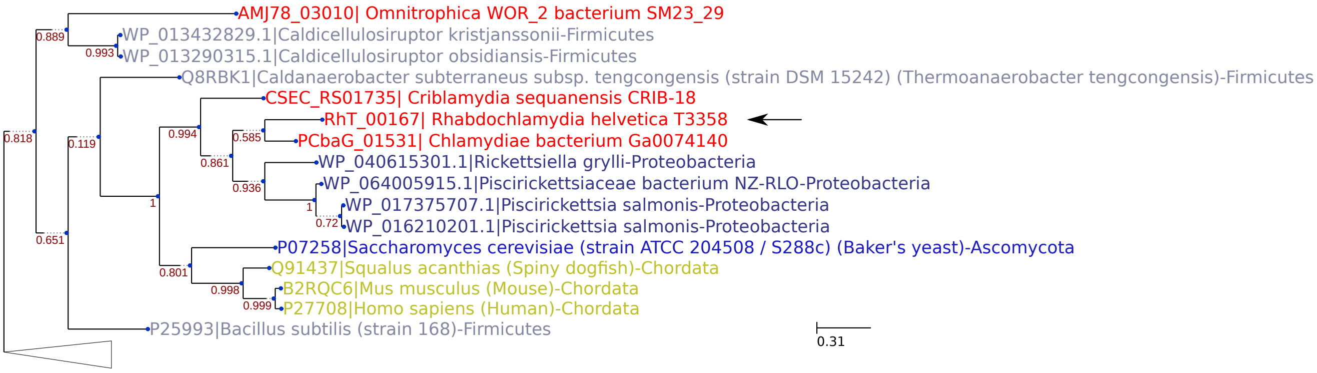

Figure S20: Phylogeny of *carA* including closest identified RefSeq homologs.

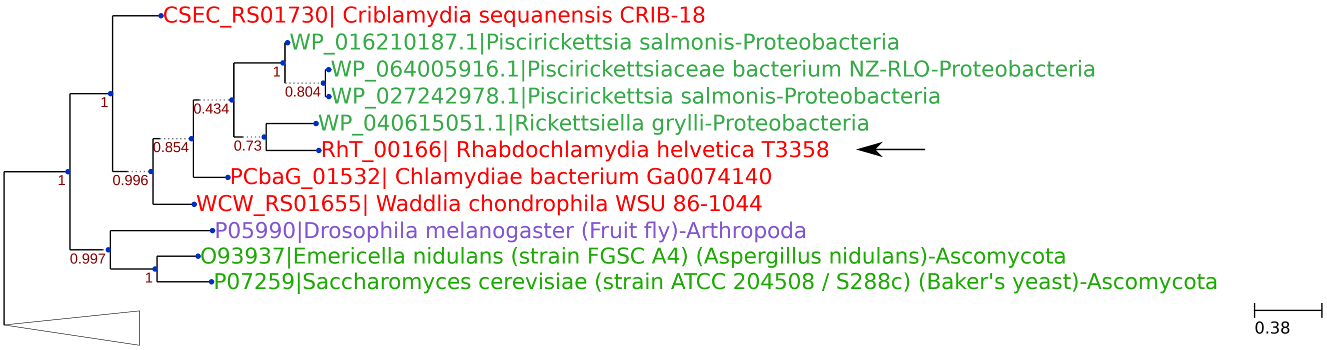

Figure S21: Phylogeny of *carB* including closest identified RefSeq homologs.
